# Supplementary figures and images for: Artificial intelligence-guided discovery of gastric cancer continuum
Source: Gastric Cancer. 2023 Jan 24;26(2):286–97. doi: 10.1007/s10120-022-01360-3 (PMC9871434; doi:10.1007/s10120-022-01360-3)

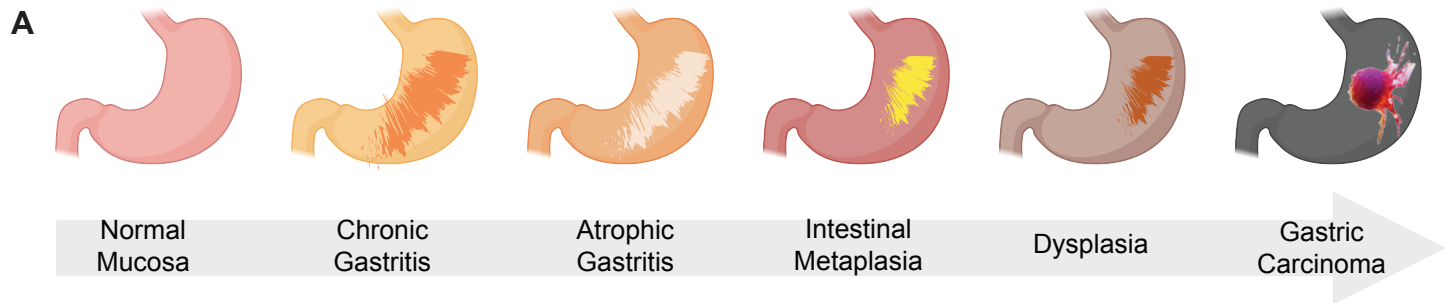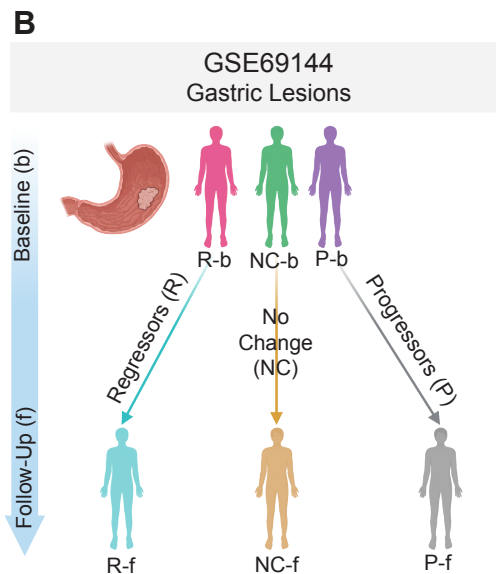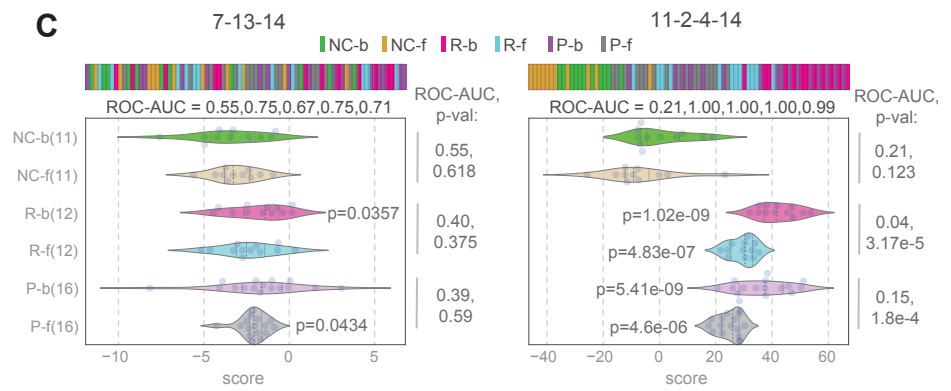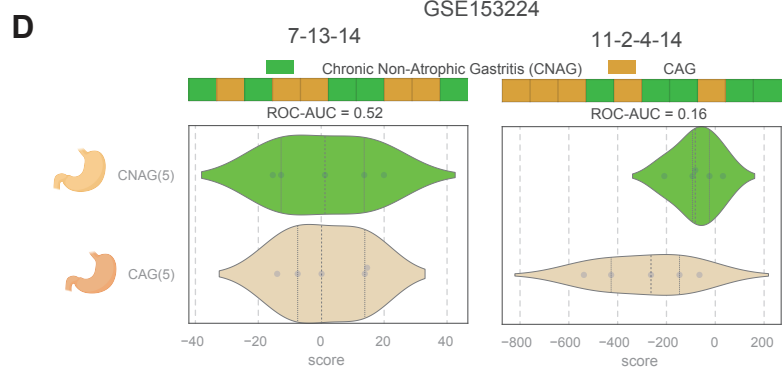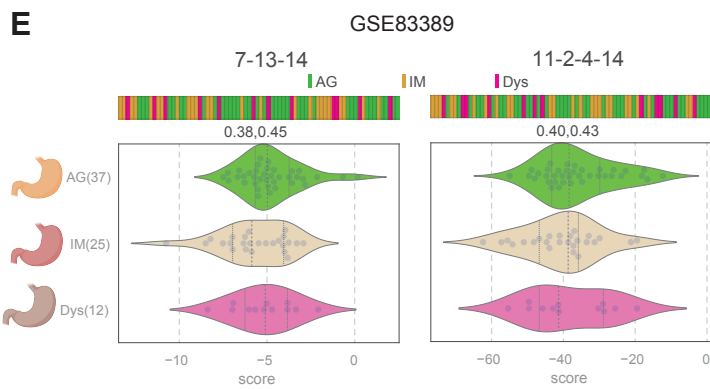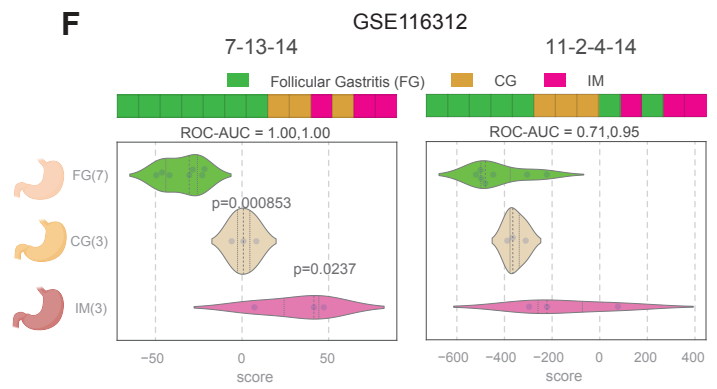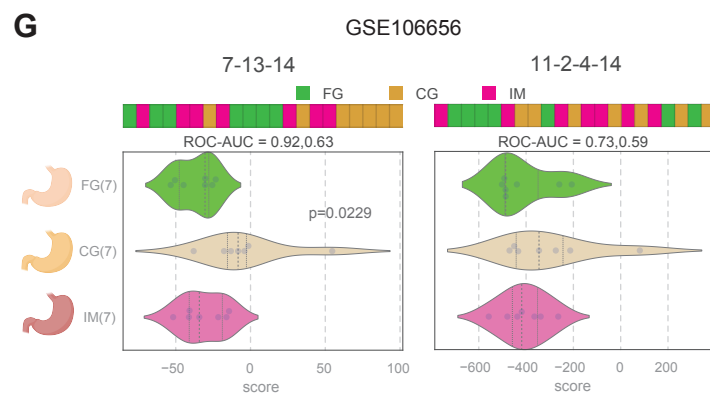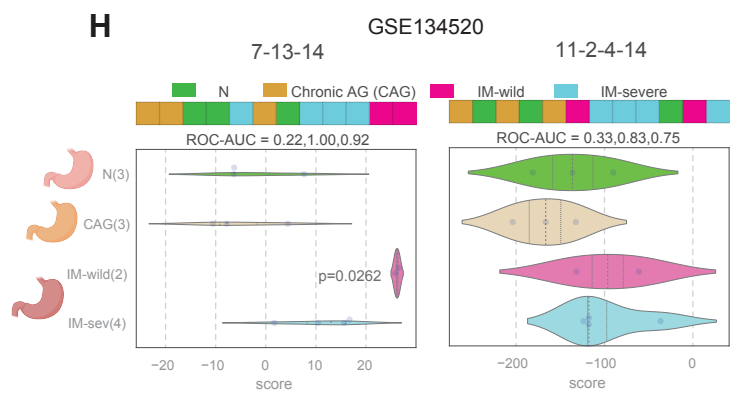

Supplement: Supplementary file 5 — Supplementary Online Resource 5 Analysis of atrophic gastritis datasets using GC-BoNE model. a. Schematic hypothetical disease continuum path from normal, gastritis, intestinal metaplasia to gastric carcinoma. b. Schematic describing study in GSE69144. c-h. Violin plots for gastritis datasets (GSE69144, GSE153224, GSE83389, GSE116312, GSE106656, GSE134520) using the GC-BoNE signature: 11-2-4-14 (left) and 7-13-14 (right) file5 (PDF 3082 KB) [file 10120_2022_1360_MOESM5_ESM.pdf]
