# Supplementary material for: Artificial intelligence-guided discovery of gastric cancer continuum
Source: Gastric Cancer. 2023 Jan 24;26(2):286–97. doi: 10.1007/s10120-022-01360-3 (PMC9871434; doi:10.1007/s10120-022-01360-3)
Supplement: Supplementary file 4 — Supplementary Online Resource 4 Bubble plots of ROC-AUC values (radius of circles is based on the ROC-AUC) demonstrating the direction of gene regulation (Up: red, Down: blue) for the classification of samples in 38 mouse models (GC-BoNE clusters in columns; sample comparison in rows). P-values based on Welch’s T-test (of composite score of gene expression values) are provided using the standard code (*p<=0.05, **p<=0.01, ***p<=0.001) next to the ROC-AUC file4 (PDF 565 KB) [file 10120_2022_1360_MOESM4_ESM.pdf]

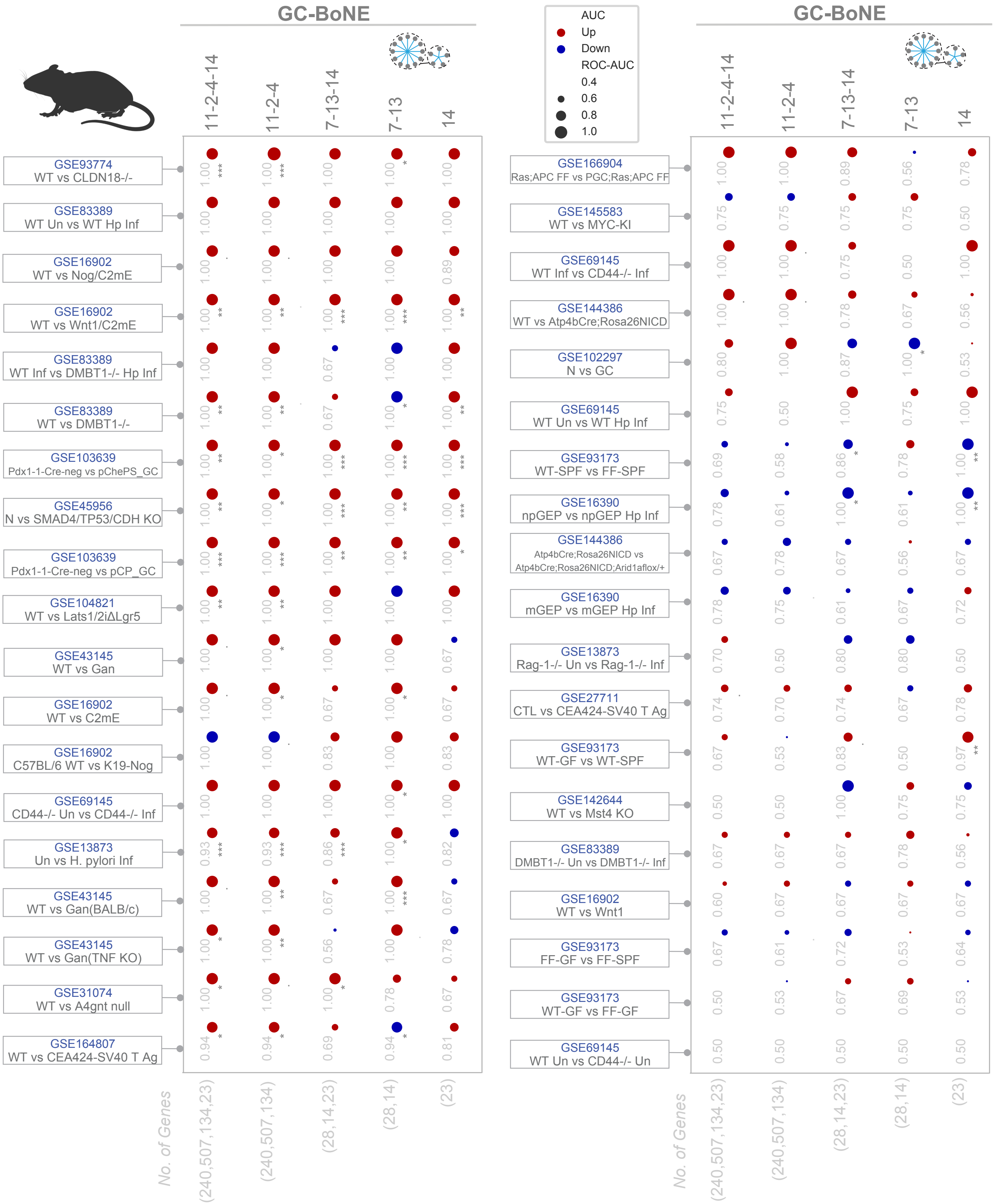
GC-BoNE
AUC
Up
Down
ROC-AUC
0.4
0.6
0.8
1.0
11-2-4-14
11-2-4
7-13-14
7-13
14
GSE93774
WT vs CLDN18-/-
GSE83389
WT Un vs WT Hp Inf
GSE16902
WT vs Nog/C2mE
GSE16902
WT vs Wnt1/C2mE
GSE83389
WT Inf vs DMBT1-/- Hp Inf
GSE83389
WT vs DMBT1-/-
GSE103639
Pdx1-1-Cre-neg vs pChePS_GC
GSE45956
N vs SMAD4/TP53/CDH KO
GSE103639
Pdx1-1-Cre-neg vs pCP_GC
GSE104821
WT vs Lats1/2iΔLgr5
GSE43145
WT vs Gan
GSE16902
WT vs C2mE
GSE16902
C57BL/6 WT vs K19-Nog
GSE69145
CD44-/- Un vs CD44-/- Inf
GSE13873
Un vs H. pylori Inf
GSE43145
WT vs Gan(BALB/c)
GSE43145
WT vs Gan(TNF KO)
GSE31074
WT vs A4gnt null
GSE164807
WT vs CEA424-SV40 T Ag
No. of Genes
(240,507,134,23)
(240,507,134)
(28,14,23)
(28,14)
(23)
GC-BoNE
11-2-4-14
11-2-4
7-13-14
7-13
14
GSE166904
Ras;APC FF vs PGC;Ras;APC FF
GSE145583
WT vs MYC-KI
GSE69145
WT Inf vs CD44-/- Inf
GSE144386
WT vs Atp4bCre;Rosa26NICD
GSE102297
N vs GC
GSE69145
WT Un vs WT Hp Inf
GSE93173
WT-SPF vs FF-SPF
GSE16390
npGEP vs npGEP Hp Inf
GSE144386
Atp4bCre;Rosa26NICD vs
Atp4bCre;Rosa26NICD;Arid1aflox/+
GSE16390
mGEP vs mGEP Hp Inf
GSE13873
Rag-1-/- Un vs Rag-1-/- Inf
GSE27711
CTL vs CEA424-SV40 T Ag
GSE93173
WT-GF vs WT-SPF
GSE142644
WT vs Mst4 KO
GSE83389
DMBT1-/- Un vs DMBT1-/- Inf
GSE16902
WT vs Wnt1
GSE93173
FF-GF vs FF-SPF
GSE93173
WT-GF vs FF-GF
GSE69145
WT Un vs CD44-/- Un
No. of Genes
(240,507,134,23)
(240,507,134)
(28,14,23)
(28,14)
(23)
